# Supplementary material for: Splice-Junction-Based Mapping of Alternative Isoforms in the Human Proteome
Source: Cell Rep. Author manuscript; Available in PMC 2020 Jan 15. (PMC6961840; doi:10.1016/j.celrep.2019.11.026)
Supplement: 2 [file NIHMS1546469-supplement-2.zip › DF1/PXD006675/CardiacFibroblast/CardiacFibroblast_18_SLC25A11_IQAVLTGLR.pdf]

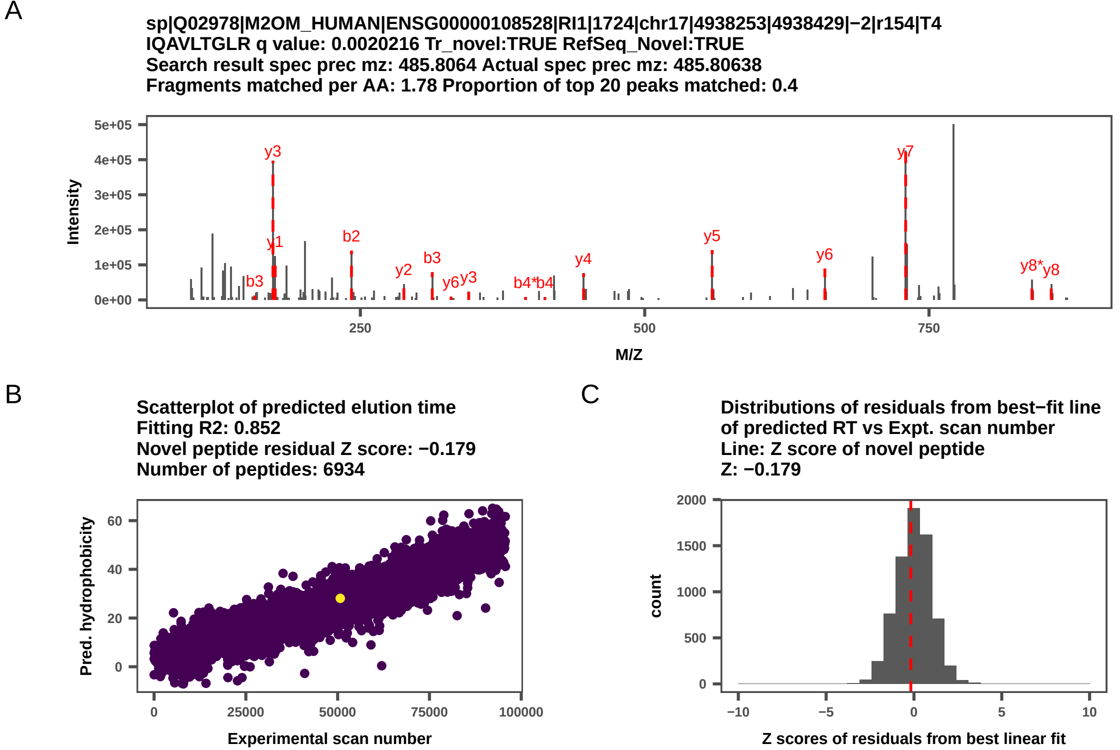

A
sp|Q02978|M2OM_HUMAN|ENSG00000108528|RI1|1724|chr17|4938253|4938429|−2|r154|T4
IQAVLTGLR q value: 0.0020216 Tr_novel:TRUE RefSeq_Novel:TRUE
Search result spec prec mz: 485.8064 Actual spec prec mz: 485.80638
Fragments matched per AA: 1.78 Proportion of top 20 peaks matched: 0.4
Intensity
5e+05
4e+05
3e+05
2e+05
1e+05
0e+00
y3
y1
b3
b2
y2
b3
y6
y3
b4*
b4
y4
y5
y6
y7
y8*
y8
250
500
750
M/Z
B
Scatterplot of predicted elution time
Fitting R2: 0.852
Novel peptide residual Z score: −0.179
Number of peptides: 6934
Pred. hydrophobicity
60
40
20
0
0
25000
50000
75000
100000
Experimental scan number
C
Distributions of residuals from best−fit line
of predicted RT vs Expt. scan number
Line: Z score of novel peptide
Z: −0.179
count
2000
1500
1000
500
0
−10
−5
0
5
10
Z scores of residuals from best linear fit
